# Geographical genetic structure of *Schistosoma japonicum* revealed by analysis of mitochondrial DNA and microsatellite markers

**DOI:** 10.1186/s13071-015-0757-x

**Published:** 2015-03-08

**Authors:** Mingbo Yin, Hongyan Li, Donald P McManus, David Blair, Jing Su, Zhong Yang, Bin Xu, Zheng Feng, Wei Hu

**Affiliations:** School of Life Science, Fudan University, Handan Road 220, Shanghai, 200433 China; National Institute of Parasitic Diseases, Chinese Center for Disease Control and Prevention, 207 Rui Jin Er Road, Shanghai, 200025 China; QIMR Berghofer Medical Research Institute, 300 Herston Road, Brisbane, Qld 4029 Australia; School of Marine and Tropical Biology, James Cook University, Townsville, Qld 4811 Australia

**Keywords:** *Schistosoma japonicum*, Genetic diversity, Genetic structure, Population differentiation, Mitochondrial DNA, Microsatellite

## Abstract

**Background:**

*Schistosoma japonicum* is a significant public health risk in parts of China and elsewhere in Southeast Asia. To gain an insight into the epidemiology of schistosomiasis japonica, a detailed investigation of *S. japonicum* genetic population structure is needed.

**Methods:**

Using three mitochondrial DNA fragments and ten microsatellite loci, we investigated the genetic diversity within and structure among twelve populations of *S. japonicum* sampled on a geographical scale covering most major endemic areas.

**Results:**

*Schistosoma japonicum* lineages from Indonesia, the Philippines and Chinese Taiwan were clearly distinct from each other and from those in mainland China. Within mainland China, there was some evidence for genetic divergence between populations from the mountain and lake regions. However, the analysis inferred no clear sub-population structure in the lake region of mainland China. High genetic diversity was found among *S. japonicum* populations of mainland China and this was significantly higher than those from island regions.

**Conclusions:**

High genetic diversity within and substantial differentiation among populations were demonstrated in *S. japonicum*.

**Electronic supplementary material:**

The online version of this article (doi:10.1186/s13071-015-0757-x) contains supplementary material, which is available to authorized users.

## Background

Schistosomiasis is one of the most serious of human parasitic diseases, infecting an estimated 200 million people in 76 tropical and subtropical countries [1]. Three major species, *Schistosoma japonicum*, *S. mansoni* and *S. haematobium*, can infect humans [2]. Of these, *S. mansoni* is endemic in Africa, the Middle East and South America and *S. haematobium* is found in Africa and the Middle East, whereas *S. japonicum* occurs in China, Indonesia and the Philippines. At present, this parasite is endemic in China in the lake/marshland regions of Anhui, Hubei, Hunan and Jiangxi provinces and in the mountainous areas of Sichuan and Yunnan provinces [3]. Although tremendous efforts over the last 20–30 years have reduced the prevalence of *S. japonicum* in China by more than 90% [4], recent indications are that schistosomiasis has re-emerged in certain areas over the last decade [5]. To gain an insight into the transmission and epidemiology of Asian schistosomiasis, a detailed investigation of *S. japonicum* population genetic structure is required.

Several classes of molecular markers have been applied to investigate the genetic variability of *S. japonicum* populations. Earlier studies using isoenzymes [6] and mitochondrial (mt) DNA sequences [7] reported remarkable genetic similarity between Chinese and Philippine *S. japonicum* lineages. Later, differences among, but not within, Chinese *S. japonicum* populations were detected using mt DNA sequences (*nad1*) [8]. Similarly, Gasser *et al.* [9] applied random amplified polymorphism DNA (RAPD) analysis, and found genetic variability among Chinese *S. japonicum* populations. Subsequently, a set of high-resolution microsatellite markers was used to investigate the genetic structure of *S. japonicum* populations from eight locations across China, and a high level of polymorphism was detected both between and within populations [10]. Moreover, *S. japonicum* populations from the lake and mountainous regions were genetically distinct [10]. Similar results from mt DNA data suggested that geographical separation was the major factor accounting for the population divergence of *S. japonicum* between the lake and mountainous regions in China [3,11]. Previous studies sampled *S. japonicum* either from mainland China, or a few locations in mainland China and other Asian countries [12,13], but did not explore the genetic population structure of *S. japonicum* across its entire range.

Most phylogeographical studies have relied on the analysis of mitochondrial DNA variation, because of its rapid evolution and maternal transmission without intermolecular recombination [14]. In contrast, microsatellites can provide information on relatively recent evolutionary processes [15]. Assessing the congruence among independent genetic markers has become an important issue [14]. Indeed, more and more studies have inferred genetic relationships among populations based on the combined analysis of different classes of markers [16-18]. Consistent results provide a robust perspective on species’ evolutionary history [19], while findings of incongruent patterns from different markers are also valuable to demonstrate important evolutionary processes, such as introgressive hybridization [20]. To date, no study has been undertaken on *S. japonicum* to investigate the genetic relationships among populations using different classes of genetic markers simultaneously on the same samples.

In this work, we analyzed *S. japonicum* samples from eight locations in China mainland (covering the lake and mountainous regions) and Taiwan, Indonesia, Japan and the Philippines. We explored the genetic relationships among these populations based on the analysis of three mt genes (*nad1*, *nad4* and 16 s-12 s) and ten microsatellite loci from the same individual worms.

## Methods

### Ethics statement

All procedures involving animals were carried out based on the guidelines of the Association for Assessment and Accreditation of Laboratory Animal Care International. The study protocol followed institutional ethical guidelines that were approved by the ethics committee at the National Institute of Parasitic Diseases, Chinese Center for Disease Control and Prevention (NIPD, China CDC; Permit No: IPD2008-4).

### Sample collection

**Figure 1:**
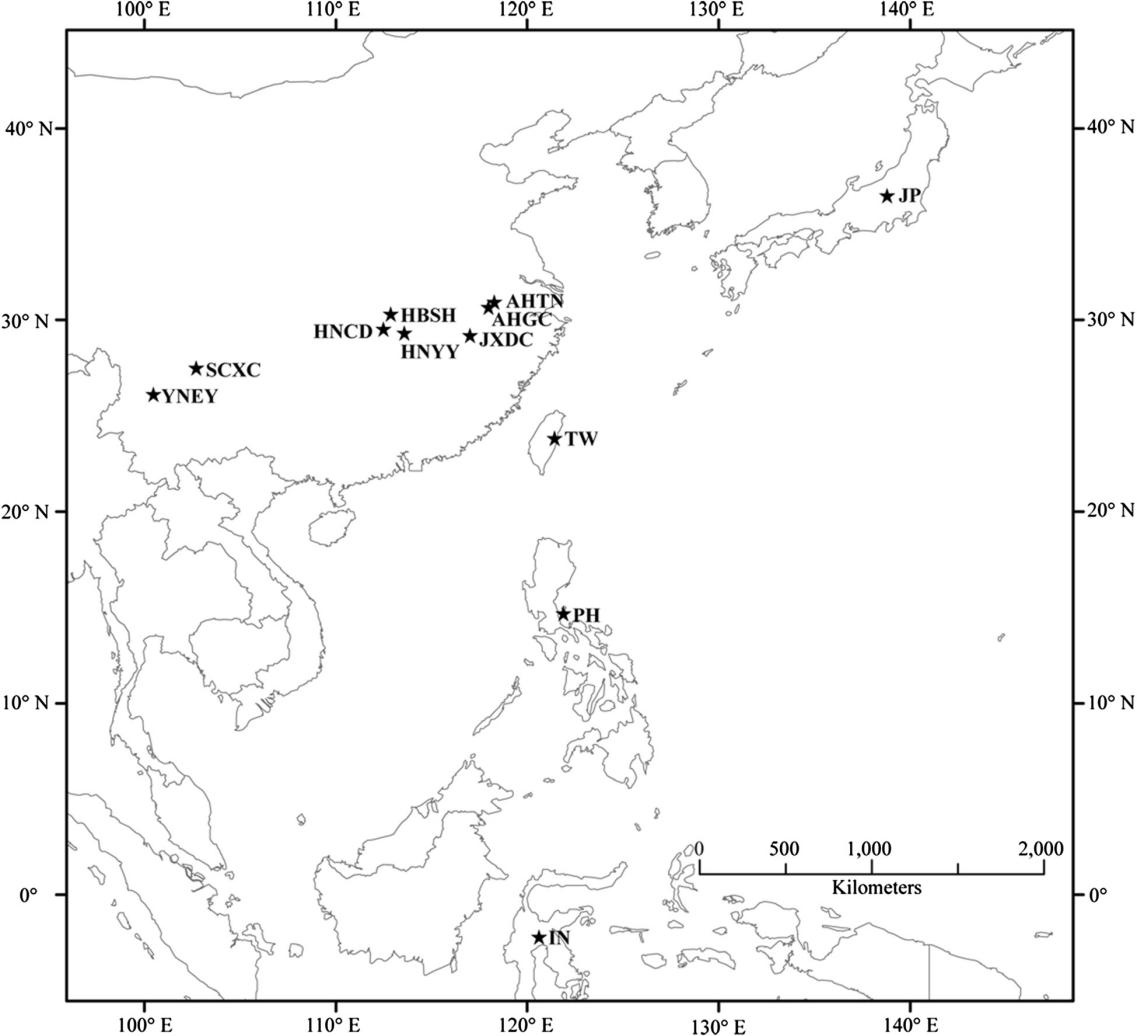
**Map showing the sampling locations of** ***S. japonicum*** **for this study.** Abbreviations: AHGC: Guichi Country, Anhui Province; AHTL: Tongling Country, Anhui Province; HBSH: Shashi City, Hubei Province; HNCD: Changde City, Hunan Province; HNYY: Yueyang City Hunan Province; JXDC: Duchang Country, Jiangxi Province; SCXC: Xichang City, Sichuan Province; YNEY: Dali City, Eryuan Country, Yunnan Province; CTW: Chinese Taiwan; IN: Indonesia JP: Japan; PH: The Philippines.

Adult individuals of *S. japonicum* were obtained from twelve different geographical locations (Figure 1), including eight locations across six provinces in mainland China (Anhui, Hunan, Hubei and Jiangxi Provinces in the Yangtze River Basin/lake region, and Sichuan and Yunnan Provinces in the mountainous area of Southwest China) and four other island locations from Asia (Taiwan, Indonesia, Japan and the Philippines). Infected snails (*Oncomelania hupensis*) from each mainland Chinese location were transported to the laboratory of NIPD, China CDC, Shanghai. Cercariae were released from pooled infected snails from each site and used to infect laboratory-raised rabbits. Finally, 45 days later, the adult schistosomes were perfused from the mesenteric veins of the infected rabbits and washed in saline before being preserved in 95% (v/v) ethanol at 4°C. The *S. japonicum* isolate from Leyte, the Philippines, was taken into culture originally in 1969 by Dr. Scholice. The material sent to us, as lyophilized adult worms, was provided by Dr. John Bruce, Centre for Tropical Diseases, University of Lowell, USA. The worms from Japan (Kofu) were gifted by Dr. Hiroshi Yamasaki, National Institute of Infectious Diseases, Tokyo, Japan. The other geographical samples (lyophilized adult worms) from Indonesia (Lindu Lake, Sulawesi) and Taiwan (Changhua) were provided by Dr. John Cross, Uniformed Services University of the Health Sciences, Bethesda, USA. These preserved samples were from populations that had been maintained in laboratory animals.

In total, 42 worms were subjected to mitochondrial DNA analysis and 486 individuals were used for the microsatellite analysis (Table 1). Microsatellite data for 401 of the worms came from our previous work [21].

**Table 1:** **Localities, sample information and genetic diversity of** ***S. japonicum*** **based on three mtDNA genes and microsatellites**

**Population location (and abbreviation used)**	**Latitude**	**Longitude**	**mtDNA genes**			**Microsatellites**							
			**N**_**1**_ *	**N**_**2**_ *	**Nucleotide diversity (π)**	**N**_**3**_ *	**N**_**4**_ *	**G**	**N**_**5**_ *	***H***_***o***_	***H***_***e***_	**HWE**	***F***_***IS***_
Anhui Province, Guichi Country, Minsheng Village (AHGC)	30.67	117.45	4	1	0	70(66)	54	52	22	0.79	0.88	***	0.13
Anhui Province, Tongling Country, Laozhou Island, Guanghui Village (AHTN)	30.94	117.76	2	2	0.0033	34(32)	27	25	23	0.82	0.89	***	0.09
Hubei Province, Shashi City, Maling Village (HBSH)	30.32	112.35	4	2	0.0001	47(43)	27	25	25	0.84	0.85	*	0.04
Hunan Province, Changde City, Wuyi Village (HNCD)	28.94	112.16	4	4	0.0050	44(40)	32	31	22	0.86	0.87	**	0.03
Hunan Province, Yueyang City, Laogang Village (HNYY)	29.34	113.07	4	4	0.0042	77(73)	55	54	45	0.79	0.91	***	0.14
Jiangxi Province, Duchang Country, Tangmei Village (JXDC)	29.20	116.50	4	3	0.0031	98(94)	68	67	71	0.80	0.90	***	0.11
Sichuan Province, Xichang City, Daxing Township, Shian and Jianxin Village combined (SCXC)	27.50	102.20	3	3	0.0012	31(28)	23	22	20	0.75	0.85	***	0.15
Yunnan Province, Dali City, Eryuan Country (YNEY)	26.12	99.95	4	4	0.0009	20	14	13	15	0.60	0.84	***	0.31
Chinese Taiwan (CTW)	23.85	120.92	5	4	0.0003	18	15	11	6	0.25	0.30	NS	0.25
Indonesia (IN)	−2.20	120.10	3	3	0.0010	18	16	9	6	0.30	0.29	NS	0.03
Japan (JP)	36.50	138.30	2	2	0.0003	4	nc	nc	4	nc	nc	nc	nc
The Philippines (PH)	14.69	121.37	3	2	0.0002	25	19	5	3	nc	nc	nc	nc

N_1_: number of individuals for mtDNA analysis; N_2_: number of mtDNA haplotypes; N_3_: number of individuals for microsatellite analysis, in parentheses is the number of individual data from previous work [21]; N_4_: number of individuals for MLG analysis excluding missing data; N_5_: number of individuals for population genetic analysis after removing near-identical MLGs; G, number of MLGs; nc, not calculated because of small sample size; *: P < 0.05; **: P < 0.01; ***: P < 0.001; NS: no significance.

### DNA extraction

Genomic DNA was extracted from individual adult schistosomes using the DNeasy Blood & Tissue Kit and Animal Tissues (Spin-Column) protocol from QIAGEN (Hilden, Germany), and stored at −20°C until use.

### Mitochondrial gene amplification and sequencing

We amplified three fragments of the mitochondrial genome: 891 bp of the gene encoding NADH dehydrogenase subunit 1 (*nad1*), 1275 bp of NADH dehydrogenase subunit 4 (*nad4*) and 1748 bp of 16S-12S mitochondrial ribosomal DNA (16S-12S rRNA). Three pairs of primers were designed based on the complete reference mitochondrial sequence for *S. japonicum* (ID in GenBank: AF215860) as follows:

*nad1*-F (5′-TTGGAGTTTGTCAGGCTTTAGG),

*nad1*-R (5′-TATATCAAACACCATCAAAAGGAAC),

*nad4*-F (5′-TGCTGTTTTATGCTATGCTACGAAG),

*nad4*-R (5′-TACAAACGGACGGACCAATAAAAC),

16S-12S rRNA-F (5′-ATAATGTTGCGTCTAAGGTC)

and 16S-12S rRNA-R (5′-TAAACACTACCCATCAAATC).

The amplifications were performed in 20 μl reactions containing 0.5 μM of each primer, ~ 50 ng of genomic DNA from an individual worm, Has *Taq* polymerase (1.25 U, TaKaRa), 1.25 mM MgCl_2_, 2 μl 10× reaction buffer and 1 μl dNTPs (2.5 mM, TaKaRa). The PCR protocol used a hot-start activation at 94°C for 10 min, followed by 35 cycles of denaturation for 30 s at 94°C, 30 s annealing (55°C for *nad1* and *nad4*, 49°C for 16S-12S rRNA) and extension (90 s for *nad1*, 150 s for *nad4* and 16S-12S rRNA) at 72°C, followed by a final extension at 72°C for 10 min. PCR products were examined using agarose gel electrophoresis (1% w/v) to validate amplification efficiency. Amplification products were then sent to BGI (Beijing Genome Institute; Shanghai, China) for sequencing using an ABI 3730 DNA Analyzer.

### Microsatellite genotyping

The DNA of each individual *S. japonicum* worm was genotyped at ten microsatellite loci (i.e. Sjp1, 4, 5, 6, 8, 9, 10, 14, 15, and 17), which were characterized previously [21]. The PCRs were performed based on the protocol described in Yin *et al.* [21]. The PCR products were diluted to an appropriate concentration and analyzed on an ABI 3730 capillary automated sequencer using a LIZ 500 labeled size standard. The allele sizes were read using GeneMapper software (Version 4.0) and checked manually.

### Mitochondrial DNA analysis

Unique haplotypes for the mitochondrial DNA sequences (concatenated *nad1*, *nad4* and 16S-12S rRNA sequences; hereafter “combined mtDNAs”) were identified using Arlequin 3.11 [22]. All unique haplotypes were aligned in ClustalW [23]. A phylogenetic tree was constructed using three methods, namely neighbor-joining (NJ), maximum-likelihood (ML) and maximum parsimony (MP), in MEGA 5.10 [23]. A consensus tree was obtained after bootstrap analysis using 10^4^ bootstrap replicates. *Schistosoma mansoni* (ID in GenBank: NC_002545.1) was used as an outgroup. A median-joining network was generated to infer the relationships among the haplotypes of *S. japonicum* using Network [24]. Nucleotide diversity (π) per population was calculated in DnaSP Version 5 [25]: comparisons between regions (mainland China *vs* island region and lake *vs* mountainous regions in mainland China) were made using unpaired Student’s *t* test. Hierarchical analysis of molecular variance (AMOVA) was applied to partition the genetic variances into among regions (i.e. mountain and lake regions), among populations within region and within populations, for samples from mainland China, in Arlequin.

### Microsatellite analysis

Multiple genotypes from a single snail are often very similar to one another and possibly derived from a single miracidium by mutations occurring during the proliferation of cercariae inside the snail [21]. We therefore retained only a single individual from each cluster of near-identical Multi Locus Genotypes (near-identical MLGs) which formed tight single-sex clusters in principal-coordinates analyses (PCoAs). After this, the PCoA was applied to explore the relationships among remaining MLGs. For each population, the genetic diversity was also examined by calculating the expected heterozygosity (*H*_*e*_) and observed heterozygosity (*H*_*o*_) in GenAIEx. In this and following analyses, the individuals for which data were missing were excluded, as were populations with N < 6. Third, the inbreeding coefficient (*F*_*IS*_), which measures the extent of nonrandom mating, was computed in Genepop 3 [26]. Positive values of *F*_*IS*_ indicate heterozygote deficiency, whereas, negative *F*_*IS*_ values suggest heterozygote excess. Finally, deviations from Hardy-Weinberg equilibrium (HWE) were tested in Genepop, with 10^4^ permutations.

Genetic population differentiation was estimated using Wright’s F-statistics (*F*_*ST*_) in GenAIEx, and the significance of the *F*_*ST*_ value was tested by 10^4^ permutations. AMOVA was applied to quantify intra- and inter-population and region variances in Arlequin. A Mantel test was used to test whether there was a relationship between pairwise geographical distance (km) and pairwise genetic distance (*F*_*ST*_, based on microsatellites) in GenAIEx.

We used STRUCTURE 2.3.4 [27] to cluster the samples on the basis of their microsatellite genotypes. The most likely number of subpopulations was estimated from the same dataset by running the procedure with a value of K from 1 to 13 using the admixture model [28]. In this process, 10^6^ iterations were performed after a burn-in of length 10^6^.

## Results

### Mitochondrial DNA analysis

We successfully amplified and sequenced 42 *S. japonicum* individuals for three complete mitochondrial fragments (*nad1*, *nad4* and 16S-12S rRNA). All the sequences were submitted to GenBank under accession numbers KP793743- KP793784 for *nad1*, KP793785-KP793826 for *nad4*, KP793827-KP793868 for 16S rRNA and KP793869-KP793910 for 12S rRNA.

**Figure 2:**
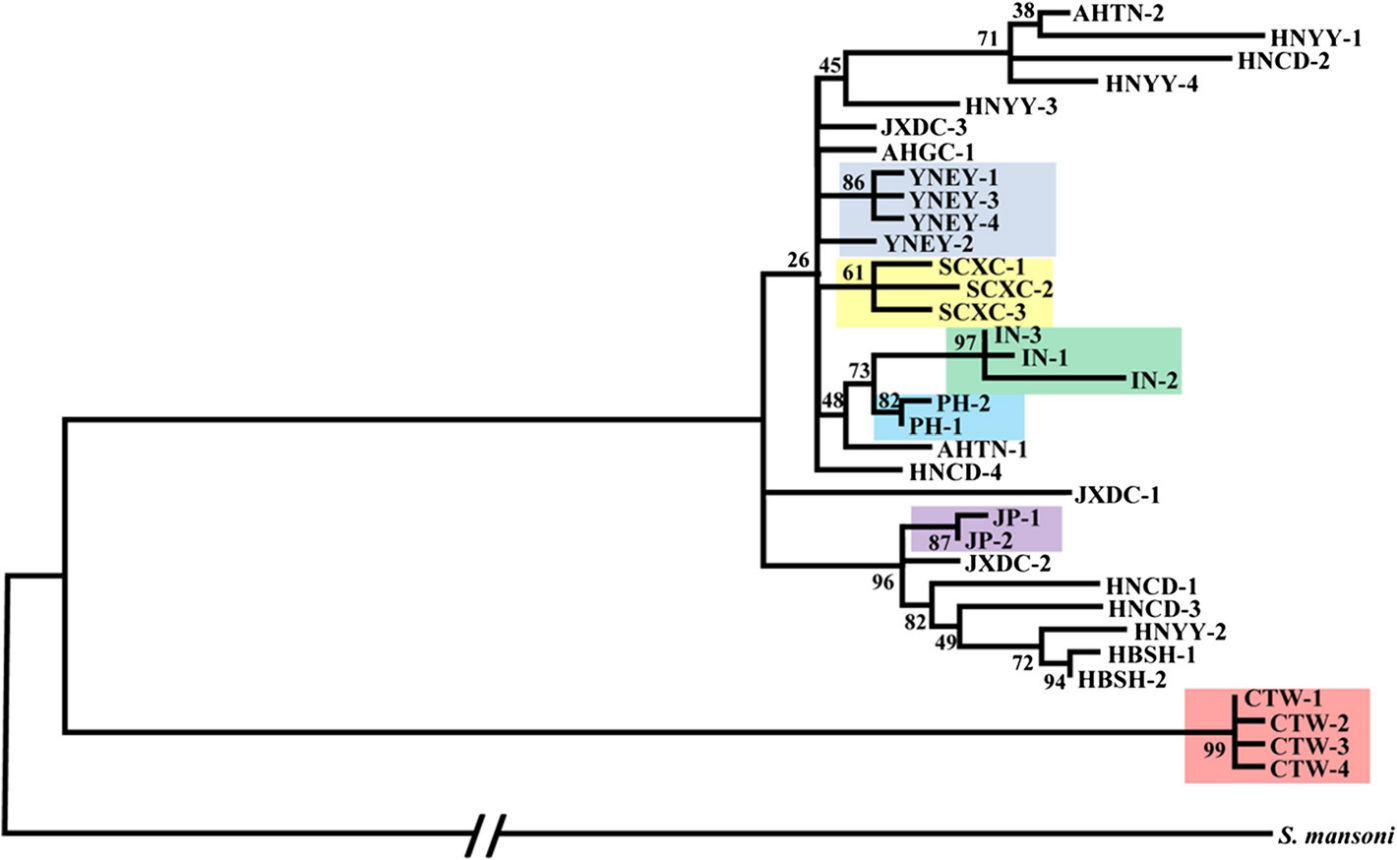
**Maximum-likelihood tree showing phylogenetic relationships of** ***S. japonicum*** **mitochondrial haplotypes (concatenated** ***nad1*** **+** ***nad4*** **+ 16S-12S rRNA).** Abbreviations: AHGC: Guichi Country, Anhui Province; AHTL: Tongling Country, Anhui Province; HBSH: Shashi City, Hubei Province; HNCD: Changde City, Hunan Province; HNYY: Yueyang City Hunan Province; JXDC: Duchang Country, Jiangxi Province; SCXC: Xichang City, Sichuan Province; YNEY: Dali City, Eryuan Country, Yunnan Province; CTW: Chinese Taiwan; IN: Indonesia JP: Japan; PH: The Philippines.

Among the 42 samples sequenced, 34 haplotypes were identified (Table 1). No haplotype was found at more than one geographical location. Phylogenetic trees, constructed by different methods (i.e. NJ, ML, and MP), showed identical or similar topologies with only a few differences in bootstrap values (not shown). The Taiwanese sequences formed a group very distant from the others. Lineages from the mountainous region of China (Sichuan and Yunnan) and the other island locations (Indonesia, the Philippines and Japan) were each distinct, forming monophletic clades (almost so in the case of Yunnan) supported by relatively high bootstrap values (Figure 2). In contrast, there was no obvious structure for samples from the lake region in China, with samples from Anhui, Hunan and Jiangxi intermingled across the tree (Figure 2). This pattern was further supported by the median-joining network (Additional file 1: Figure S1).

The nucleotide diversity (π) ranged from 0 to 0.005 (mean = 0.0016). Interestingly, the nucleotide diversities of most (five of eight) populations from mainland China were significantly higher than those from Chinese Taiwan and each of the other island regions (mean for mainland China 0.0022 *vs* 0.0005; N = 12, t = 2.52, *P* = 0.03). However, we detected no significant differences in diversity between populations from the mountainous area and lake region of mainland China (N = 8, t = 1.80, *P* = 0.13). We recognize that sample sizes are small and this might influence the results. The four individuals from Anhui Guichi shared the same haplotype. In a further seven populations (Anhui Tonglin, Hunan Changde, Hunan Yueyang, Sichuan Xichang, Yunnan Eryuan, Indonesia and Japan), each individual had a unique haplotype (Table 1). The AMOVA revealed that most of the genetic variation was at the within-population level (62.53%), however, significant genetic differentiation was found between the mountain and lake regions (6.25%; *P* < 0.001).

### Microsatellite analysis

**Figure 3:**
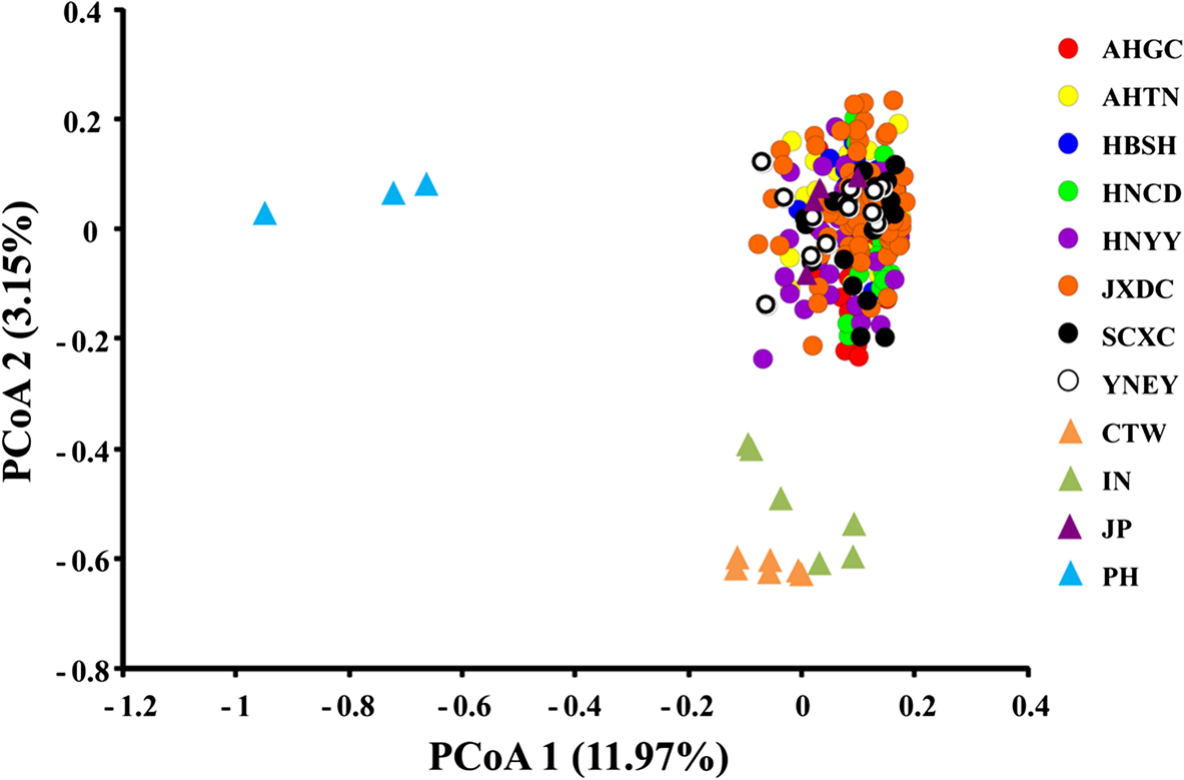
**Principal coordinates analysis (first two factors only) using 10 microsatellite loci based on co-dominant genotypic distances of all** ***S. japonicum*** **individuals (after removal of near-identical genotypes).**

At the 10 microsatellite loci, 317 unique MLGs were detected from 353 individuals (individuals with missing data were excluded: Table 1), and no MLG was shared among populations. According to the PCoA, the MLGs of 262 worms (after exclusion of near-identical MLGs) could be divided into three groups: individuals from the Philippines, Indonesia and Chinese Taiwan were clustered according to their geographical origins and all the worms from mainland China and Japan were grouped together (Figure 3). Identical results were obtained when the PCoA was run using only the 42 individuals for which mitochondrial sequence data were also obtained (not shown). Observed heterozygosity (*H*_*o*_) was consistently lower than the expected heterozygosity (*H*_*e*_), and most of the inbreeding coefficient (F_*IS*_) values were positive, with the exception of Indonesia and Japan (Table 1), indicating an excess of homozygotes within subpopulations in comparison to HWE. Indeed, all populations deviated significantly from HWE (Table 1).

**Figure 4:**
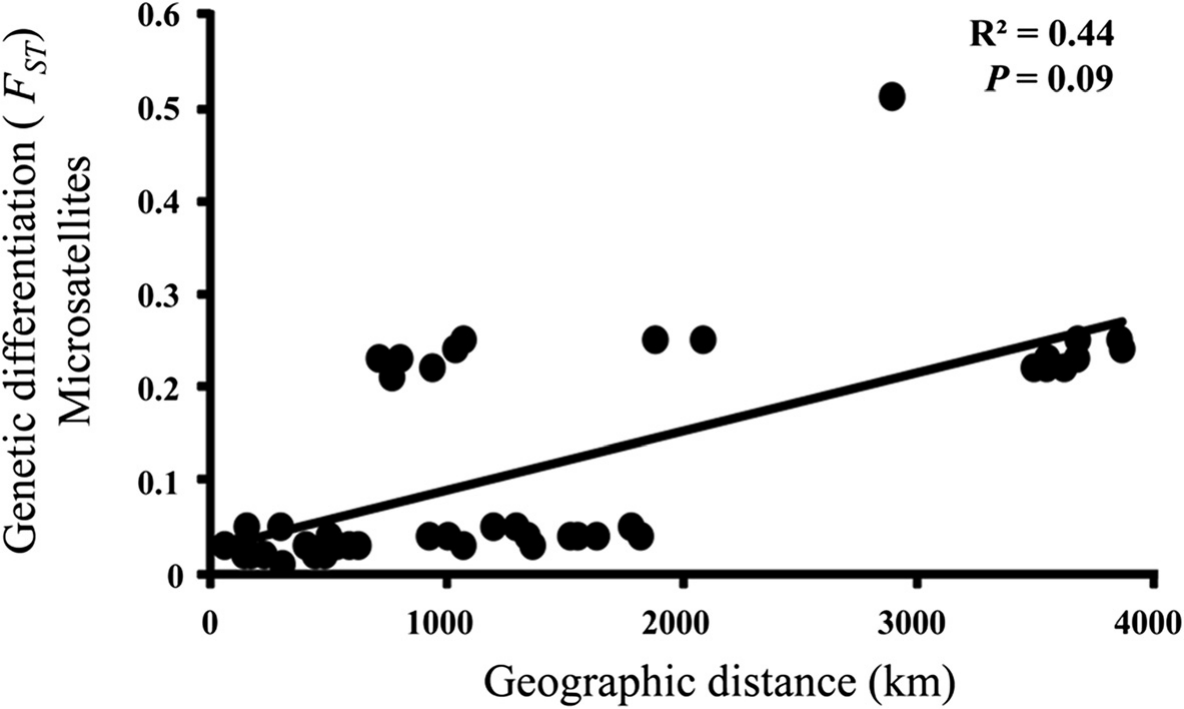
**Linear regression of genetic differentiation (** ***F***_***ST***_ **) based on ten microsatellite loci versus geographical distance between** ***S. japonicum*** **populations.**

Although most of the genetic variation occurred at the within-population level (74.91%) according to hierarchical AMOVA, significant genetic differentiation was detected among the populations. Pairwise *F*_*ST*_ values ranged from 0.01 to 0.51 (averaged over 10 loci; Table 2), suggesting low to high population differentiation. Specifically, the *F*_*ST*_ between the Taiwanese and Indonesian populations was 0.51 (Table 2), indicating high population differentiation. In contrast, relatively low differentiation was seen among the populations from mainland China (*F*_*ST*_ ranged from 0.01 to 0.05; mean = 0.03; Table 2). The Mantel test for isolation by distance revealed a positive, but non-significant, correlation between population genetic differentiation (*F*_*ST*_) based on the microsatellite data and geographical distance (*R*^*2*^ = 0.44; *P* = 0.09; Figure 4).

**Table 2:** **Pairwise genetic differentiation (** ***F***_***ST***_ **) among populations based on 10 microsatellite loci**

	**AHGC**	**AHTN**	**HBSH**	**HNCD**	**HNYY**	**JXDC**	**SCXC**	**YNEY**	**CTW**	**IN**
AHGC										
AHTN	0.03									
HBSH	0.04	0.03								
HNCD	0.03	0.03	0.05							
HNYY	0.02	0.02	0.03	0.02						
JXDC	0.02	0.02	0.03	0.02	0.01					
SCXC	0.04	0.04	0.04	0.04	0.03	0.03				
YNEY	0.05	0.04	0.05	0.05	0.04	0.04	0.05			
CTW	0.21	0.23	0.25	0.24	0.22	0.23	0.25	0.25		
IN	0.22	0.23	0.25	0.23	0.22	0.22	0.24	0.25	0.51	

Abbreviations: AHGC: Guichi Country, Anhui Province; AHTL: Tongling Country, Anhui Province; HBSH: Shashi City, Hubei Province; HNCD: Changde City, Hunan Province; HNYY: Yueyang City Hunan Province; JXDC: Duchang Country, Jiangxi Province; SCXC: Xichang City, Sichuan Province; YNEY: Dali City, Eryuan Country, Yunnan Province; CTW: Chinese Taiwan; IN: Indonesia JP: Japan. All values are significant below the level of 0.05 (based on 10^4^ permutations).

**Figure 5:**
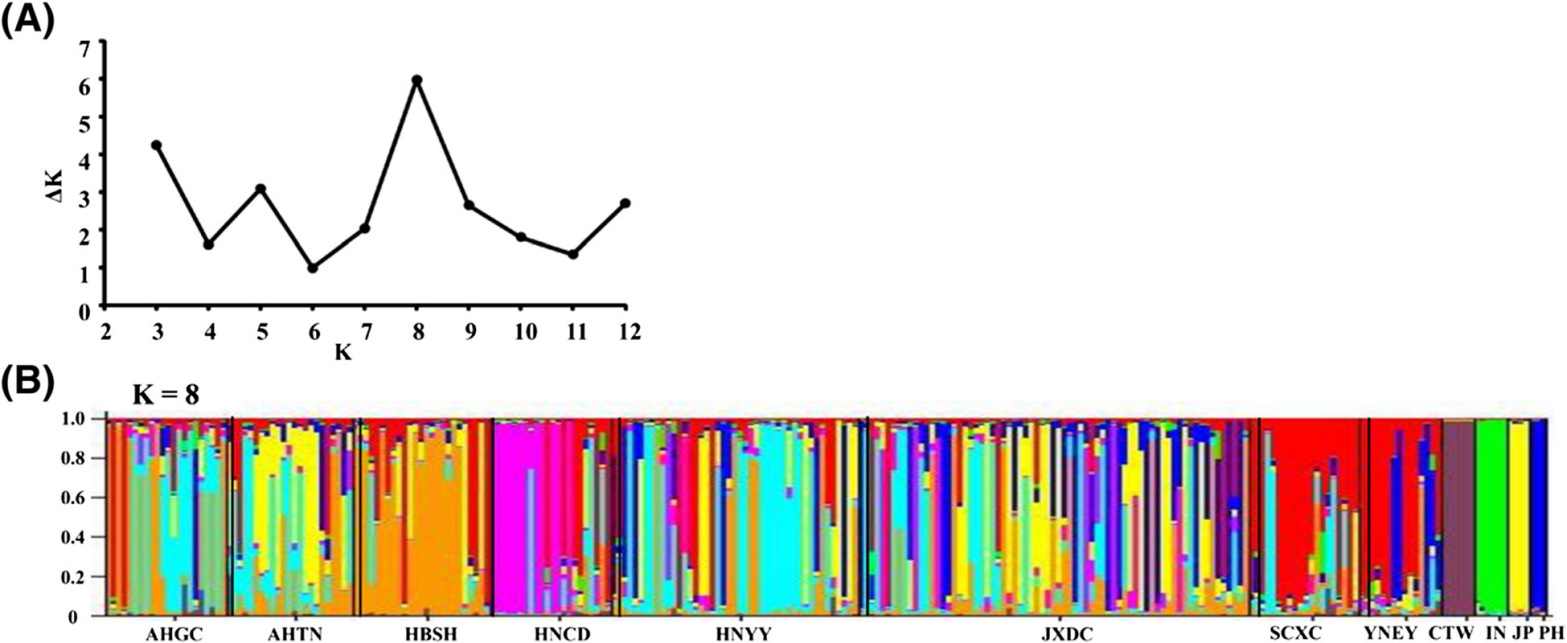
**Assignment of specimens of** ***S. japonicum*** **to populations by the STRUCTURE program. (A)** The peak of ΔK represents the most likely number of subpopulations; **(B)** Bayesian clustering results inferred by STRUCTURE with the most probable model (K = 8).

Based on the highest value of ΔK, the most likely number (K) of clusters was eight (Figure 5A). Using K = 8 in STRUCTURE, we found that samples from Indonesia, the Philippines and Taiwan each formed a unique, distinct cluster. On the other hand, lineages from mainland China and Japan were difficult to separate (Figure 5B). Other values of K were explored, without providing any additional insights (not shown). This clustering was consistent with the PCoA (Figure 3), i.e., both analyses recognized distinct lineages from Indonesia, the Philippines and Taiwan, but not within mainland China and Japan. Populations from mountain regions (Sichuan and Yunnan) were not distinguished from those in the lake regions.

## Discussion

To our knowledge, this report includes the widest geographic coverage to date, surveying 12 *S. japonicum* populations in four Asian countries to assess the degree of genetic subdivision within the species.

The two classes of markers, mitochondrial DNA and microsatellites, yielded results those were not completely congruent. Based on the phylogenetic tree (constructed using three mitochondrial DNA fragments), the Taiwanese lineage obviously diverged from the others. Moreover, the lineages from the mountain regions of mainland China, Indonesia, Japan and the Philippines were distinct from each other and from populations in the lake region. However, no substructure was detected among the lineages from the Chinese lake region. In agreement with the phylogenetic analysis, the PCoA analysis, based on ten microsatellite loci, showed that individuals from the Philippines, Indonesia and Chinese Taiwan formed distinct clusters according to their geographical origins. This pattern was further supported by a Bayesian approach in STRUCTURE. The *S. japonicum* lineages from the mountain and lake regions of mainland China and from Japan, that were distinct according to the mitochondrial DNA markers, could not be separated by the microsatellite analysis.

Although some earlier studies failed to detect any evidence of major genetic dissimilarities at the DNA level between Chinese and Philippine *S. japonicum* [7], our study, in agreement with the more recent studies using microsatellites [10], has shown that schistosome populations in the two countries are genetically distinct. Moreover, although *S. japonicum* was first described from Taiwan in 1914 [29], there have been few studies investigating genetic variability in this population [30]. Here, we provide the first report that the Taiwanese population is clearly divergent from others.

Genetic divergence observed among regional Chinese and Asian populations in this study may relate to the distribution and morphs of *Oncomelania hupensis*, the only intermediate host species complex involved in the transmission of *S. japonicum* [31]. In mainland China, there exist two distinct morphological and allozyme forms of *O. hupensis*: one with a smooth shell in the mountain region and another with a ribbed shell in the lake region [32,33]. A single phenotype of *O. hupensis quadrasi* occurs in the Philippines, while *O. h. nosophora* is present in Japan, *O. h. lindoensis* in the Indonesian islands, and *O.h. formosana* and *O. h. chiui* are present in Chinese Taiwan [34]. *Oncomelania hupensis* from China is genetically different from *O. h. quadrasi* in the Philippines [13], and the *O. quadrasi* populations in the Philippines have a substructure associated with their geographic origin [34]. These different phenotypic and genotypic morphs of *O. hupensis* that exist in different regions might contribute to the genetic divergences apparent among populations of its parasite, *S. japonicum* [35].

In the present study, *F*_*ST*_ values ranged from 0.01 to 0.51, indicating varied levels of pairwise population-genetic differentiation. In fact, strong population genetic differentiation has been detected between the mountain and lake regions of China on a large geographical scale [3,10]. This could be explained by different ecological environments and agricultural practices between the two habitat types. Another explanation may relate to the topographical isolation of the mountainous land from the lower Yangtze River basin, resulting in restrictions to gene flow. In agreement with a previous study which applied another set of microsatellite loci [36], our Mantel test showed no significant correlation between genetic and geographical distance. However, identical multilocus genotypes were not shared among the populations, which would be direct evidence of gene flow.

Corresponding well with the previous studies indicating high genetic diversity of *S. japonicum,* based on microsatellites [10] and mitochondrial DNA [3], in this study high genetic diversity was detected within most *S. japonicum* populations, according to both mitochondrial DNA and microsatellite markers. We also observed marked deviations from HWE in most populations, in agreement with findings of a previous study on *S. japonicum* [10] and similar to the results of studies on *S. mansoni* [37]. This significant deviation could have resulted from the effects of non-random mating, inbreeding and population subdivision. Also, the deviation could also have been caused by unintended non-random sampling, given the way in which specimens for analysis were obtained. Furthermore, only low numbers of individuals were available for analysis from some populations.

## Conclusions

In conclusion, high genetic diversity within and substantial genetic differentiation among *S. japonicum* populations were demonstrated using both mitochondrial DNA and microsatellites. Future research should aim to generate a comprehensive link between the population nucleotide diversity/structure differences and corresponding phenotypic variation, such as host preferences and differences in pathology, as well as possible differences in the response to anti-schistosome vaccines and immunodiagnostics. Overall, the present findings provide fundamental biological and evolutionary information on *S. japonicum*, with significant implications for the control and elimination of this parasite in Asia.

## Additional file

Additional file 1: Figure S1. Median-joining Network based on the mitochondrial haplotypes (concatenated *nad1* + *nad4* + 16-12S) of *S. japonicum.* Abbreviations: AHGC: Guichi Country, Anhui Province; AHTL: Tongling Country, Anhui Province; HBSH: Shashi City, Hubei Province; HNCD: Changde City, Hunan Province; HNYY: Yueyang City Hunan Province; JXDC: Duchang Country, Jiangxi Province; SCXC: Xichang City, Sichuan Province; YNEY: Dali City, Eryuan Country, Yunnan Province; CTW: Chinese Taiwan; IN: Indonesia JP: Japan; PH: The Philippines. The sizes of nodes are proportional to the frequencies of the haplotypes.
