# Supplementary figures and images for: Geographical genetic structure of Schistosoma japonicum revealed by analysis of mitochondrial DNA and microsatellite markers
Source: Parasit Vectors. 2015 Mar 8;8:150. doi: 10.1186/s13071-015-0757-x (PMC4372230; doi:10.1186/s13071-015-0757-x)

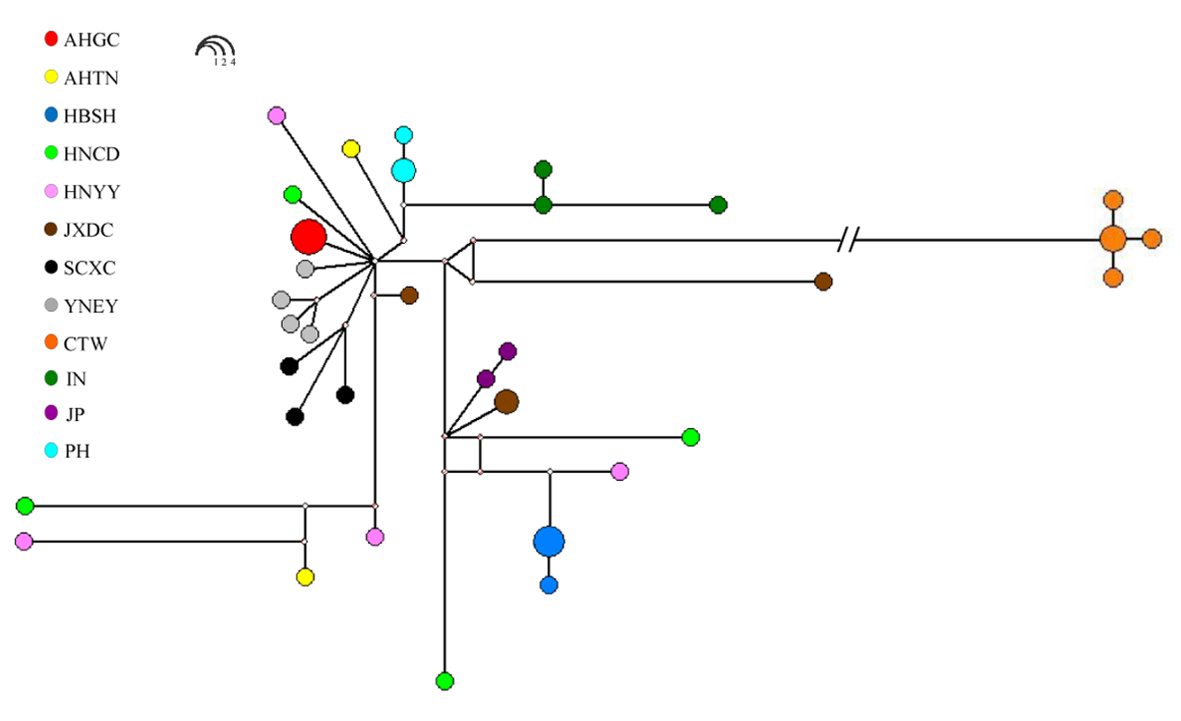

Supplement: Additional file 1: Figure S1. — Median-joining Network based on the mitochondrial haplotypes (concatenated nad1 + nad4 + 16-12S) of S. japonicum. Abbreviations: AHGC: Guichi Country, Anhui Province; AHTL: Tongling Country, Anhui Province; HBSH: Shashi City, Hubei Province; HNCD: Changde City, Hunan Province; HNYY: Yueyang City Hunan Province; JXDC: Duchang Country, Jiangxi Province; SCXC: Xichang City, Sichuan Province; YNEY: Dali City, Eryuan Country, Yunnan Province; CTW: Chinese Taiwan; IN: Indonesia JP: Japan; PH: The Philippines. The sizes of nodes are proportional to the frequencies of the haplotypes. [file 13071_2015_757_MOESM1_ESM.tif]
